# Lower Back Pain Beyond Routine Causes: Sacral Spinal Epidural Arteriovenous Fistula (SEDAVF)

**DOI:** 10.7759/cureus.64850

**Published:** 2024-07-18

**Authors:** Hari Deep Yellamilli, Srikanth Muni, Rajasekhar Rekapalli, Vemula Anjanipriya, Anuvindha JS

**Affiliations:** 1 Trauma and Emergency Medicine, All India Institute of Medical Sciences, Mangalagiri, Mangalagiri, IND; 2 Orthopaedics, All India Institute of Medical Sciences, Mangalagiri, Mangalagiri, IND; 3 Neurosurgery, All India Institute of Medical Sciences, Mangalagiri, Mangalagiri, IND; 4 Oral and Maxillofacial Surgery, All India Institute of Medical Sciences, Mangalagiri, Mangalagiri, IND

**Keywords:** digital subtraction angiography (dsa), neuro mri, arteriovenous malformation (avm), neuro-surgery, emergency medicine physician

## Abstract

Lower back pain (LBP) is a frequent complaint, even among young people. The most common cause is attributed to slipped discs or vertebral fractures. Less common etiologies should also be considered when presenting with typical symptoms of severe backache. Here, we are presenting one such uncommon case. A 32-year-old man with a history of meningocele repair in neonates presented with severe backache, urinary retention, and constipation in an emergency. Initially, the patient was treated elsewhere, but symptoms persisted. A contrast-enhanced MRI done in the emergency revealed a rare sacral spinal epidural arteriovenous fistula (SEDAVF) with cord congestion. The patient was taken up for digital subtraction angiography, which confirmed the diagnosis, and was treated successfully with endovascular embolization. This case highlights the complex presentation of SEDAVF and the importance of prompt diagnosis and intervention.

## Introduction

Back pain is a frequent complaint. The lower back pain (LBP) prevalence in young adults aged between 18 and 35 years was 42.4% per year and 22.8% per week [1]. Andersson estimated the annual worldwide LBP incidence in adults to be 15% and the point prevalence to be 30% [2]. The etiology of back pain is classified into traumatic, degenerative, oncologic, infectious, inflammatory, metabolic, referred pain, postural, congenital, and psychiatric [3]. Epidural arteriovenous fistulas comprise a major proportion of spine vascular lesions, with an annual incidence of 5-10 per million. At least 80% of patients are male, and more than 66% of patients are in the sixth and seventh decades of life, indicating a preponderance of gender and age [4]. We report a case of a 32-year-old man with severe backache and progressive lower limb sensory deficits, ultimately diagnosed with a sacral spinal epidural arteriovenous fistula (SEDAVF). Spinal arteriovenous malformations (AVMs) are rare pathologies, representing 3-4% of all space-occupying lesions affecting the spinal cord [5].

## Case presentation

A 32-year-old male with a history of meningocele repair at 10 days old presented with complaints of severe backache for 10 days, associated with an inability to pass urine and constipation. He had no history of fever, falls, trauma, or other complaints. He received treatment at another hospital a week prior with no improvement in symptoms. He has had a history of bladder incontinence since childhood and uses a self-catheter.

Examination

On arrival, the vitals were normal. A neurological examination revealed bilateral lower limb power (5/5), and he was able to walk. The straight leg-raising test was positive at 50 degrees. Sensory examination revealed diminished sensation in the lower limbs compared to the upper limbs. Local examination revealed tenderness over the L3-S1 vertebrae, normal sphincter tone, and a vertical scar on the right side of the sacrum.

Imaging

**Figure 1:**
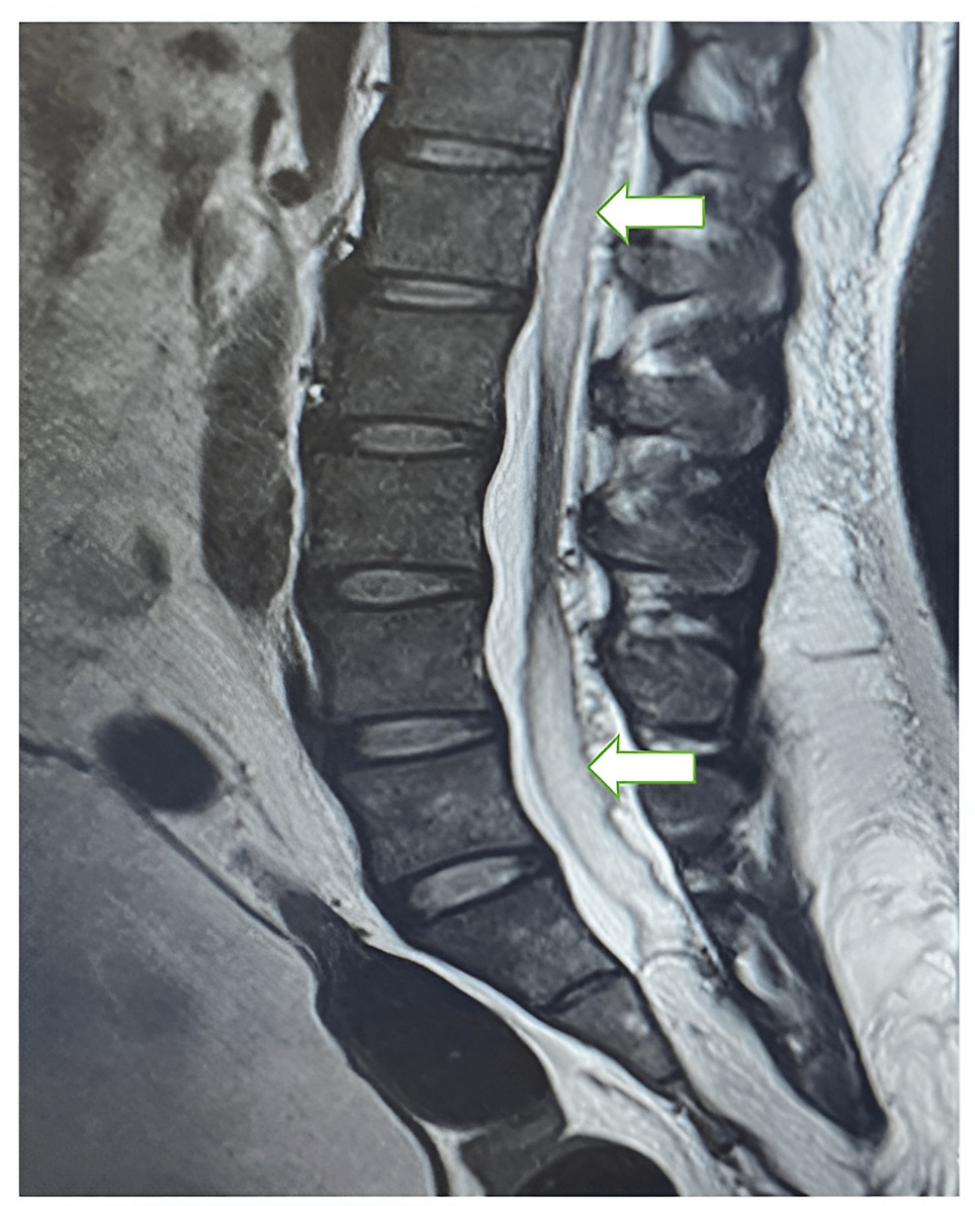
Spinal cord edema (block arrows)

The patient was taken for an emergency MRI spine with contrast, which showed sacral SEDAVF with associated posterior peri-medullary venous congestion, intermittent cord expansion, lipomyelocele, and bilateral hydroureteronephrosis. Figure 1 shows spinal cord edema with intermittent cord normal.

**Figure 2:**
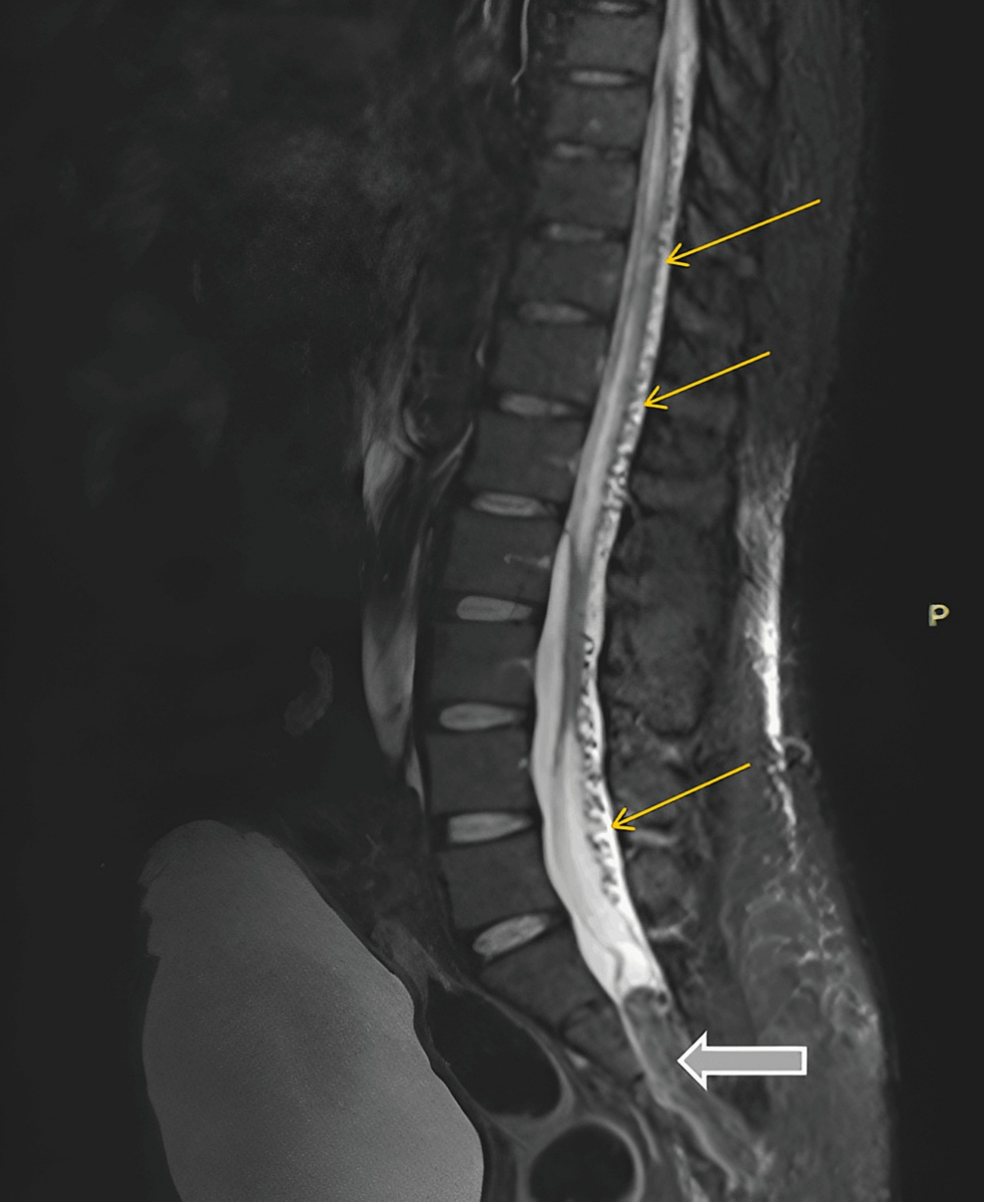
Peri-medullary congestion (small arrows) and lipomyelocele (block arrow)

Figure 2 shows the peri-medullary congestion adjacent to the spinal cord and lipomyelocele entering the spinal canal.

The patient was followed up with digital subtraction angiography (DSA), which confirmed the diagnosis and helped in the localization of the vessel. The patient underwent successful embolization of vessels with the resolution of symptoms post-procedure.

## Discussion

SEDAVFs are rare, complex lesions, often presenting with clinical manifestations secondary to compressive symptoms or congestive myelopathy. SEDAVFs are usually located in the ventral epidural space and are fed mainly by the anteriorly coursing epidural arteries [6]. The shunt flow drains first into the epidural venous sac and then to the paravertebral vein [7]. In the systematic review by Huang et al., 10% of SEDAVF patients had spine surgery prior, leading to a history of spine surgery as a risk factor for SEDAVF [8].

Clinical manifestations can vary depending on the location and size of the fistula, ranging from vague back pain and radiculopathy to progressive myelopathy and even paraplegia. DSA is regarded as a basic diagnostic tool for the angiomorphologic, pretherapeutic evaluation of spinal arteriovenous fistulas and malformations [9]. Very few indications remain for neuroangiography due to advances in CT and MRI imaging, such as intracranial aneurysms, AVMs, and dural arteriovenous shunts (both intracranially as well as in the spine), for which angiography is still necessary [10].

SEDAVFs are characterized by an epidural venous sac, usually located in the ventral epidural space, multiple bilateral feeders feeding it, and the absence of a horizontal T-sign. The T-sign is a typical sign seen in SDAVF. Kiyosue et al. found that SEAVFs frequently involve the lumbar spine (83%) [11].

Current treatment options include endovascular embolization with liquid embolic agents or coils, surgical resection of the fistula, or both. Endovascular treatment is the favored method of treatment, with about two-thirds of studies and case reports using the method. Onyx is the preferred embolic material over glue, as it is a non-thrombogenic embolic material that allows for better packing of the epidural venous sac without the risk of thrombus formation [12]. Al-Abdulwahhab et al. observed that in all 12 SEAVF patients, an epidural venous sac drains into both intradural and extradural. Spinal cord edema improved in all patients for a mean of 18 months after treatment [13]. Among the microsurgical, endovascular, and combined treatment groups of SEDAVFs, the treatment failure rate was significantly higher in the endovascular treatment group (7.5%, 31%, and 0%, respectively), according to Takai et al.'s study conducted in Japan [14]. The vast majority of SEDAVFs remain idiopathic [15].

## Conclusions

This case report highlights the typical presentation of SEDAVF with back pain and neurologic deficits. Early diagnosis and prompt intervention with endovascular embolization can significantly improve outcomes and prevent potential neurological complications in patients with spinal AVMs. Microsurgery has a lower chance of failure in treating SEDAVF.
